# Impacts of Central Administration of the Novel Peptide, LEAP-2, in Different Food Intake Models in Conscious Rats

**DOI:** 10.3390/nu16121946

**Published:** 2024-06-19

**Authors:** Chia-En Lin, Chih-Yen Chen

**Affiliations:** 1 Department of Pharmacy, Tajen University, No. 20, Weixin Rd., Yanpu Township, Pingtung County 907101, Taiwan; celin@tajen.edu.tw; 2 Division of Gastroenterology and Hepatology, Department of Medicine, Taipei Veterans General Hospital, Taipei 112201, Taiwan; 3 Institute of Emergency and Critical Medicine, and School of Medicine, National Yang Ming Chiao Tung University, Taipei 112304, Taiwan; 4 Chinese Taipei Society for the Study of Obesity, Taipei 110301, Taiwan

**Keywords:** food intake, ghrelin, ingestion behavior, liver-expressed antimicrobial peptide-2 (LEAP-2), time-restricted feeding

## Abstract

Liver-expressed antimicrobial peptide-2 (LEAP-2) has mutual antagonism with ghrelin, which evokes food intake under a freely fed state. Nevertheless, the impact of LEAP-2 on ghrelin under time-restricted feeding (TRF), which has benefits in the context of metabolic disease, is still unknown. This study aims to explore the impact of central administration of LEAP-2 on the ingestion behavior of rats, which was evaluated using their cumulative food intake in the TRF state. Before intracerebroventricular (ICV) administration of *O-n*-octanoylated ghrelin (0.1 nmol/rat), as a food-stimulatory model, the rats received various doses of LEAP-2 (0.3, 1, 3 nmol/rat, ICV). Cumulative food intake was recorded at 1, 2, 4, 8, 12, and 24 h after ICV injection under 12 h freely fed and TRF states in a light phase. In 12 h freely fed and TRF states, central administration of ghrelin alone induced feeding behavior. Pre-treatment with LEAP-2 (1 and 3 nmol/rat, ICV) suppressed ghrelin-induced food intake in a dose-dependent manner in a 12 h freely fed state instead of a TRF state, which may have disturbed the balance of ghrelin and LEAP-2. This study provides neuroendocrine-based evidence that may explain why TRF sometimes fails in fighting obesity/metabolic dysfunction-associated steatotic liver disease in clinics.

## 1. Introduction

Liver-expressed antimicrobial peptide-2 (LEAP-2) is a novel 40-amino-acid peptide recently identified as an endogenous antagonist of the ghrelin receptor (growth hormone secretagogue receptor, GHSR) [1], which is associated with metrics of the metabolic profile such as obesity [2], body mass index, liver fat content, and fasting insulin [3]. A pharmacologic approach showed that LEAP-2 and its *N*-terminal part behave as inverse agonists of GHSR and as competitive antagonists of ghrelin-induced inositol phosphate production and calcium mobilization, while the *N*-terminal region of LEAP-2 is able to inhibit ghrelin-induced food intake in mice, indicating the important role of LEAP-2 in the control of ghrelin response under both normal and pathological conditions [4,5]. LEAP-2 has been shown to be produced in the liver and small intestine, and its secretion is suppressed by fasting [1,6]. LEAP-2 fully inhibits GHSR activation by ghrelin and blocks the main effects of ghrelin *in vivo*, including promotion of food intake, GH release, and maintenance of blood glucose levels during chronic caloric restriction; however, neutralizing antibodies that block endogenous LEAP-2 function enhance the action of ghrelin *in vivo* [1]. Ghrelin is a gut hormone that is released from stomach [7,8,9,10] and acts on the hypothalamus, thus promoting feeding behavior [7]. From the above description, it can be inferred that LEAP-2 and ghrelin influence the eating behavior regulated by the hypothalamus. Taken together, LEAP-2 inhibits the central function of ghrelin through crosstalk between the liver, stomach, and brain and acts to fine-tune the action of ghrelin in response to changing environmental conditions for regulation of food intake, energy partition, and homeostasis as well as the circadian rhythm. Time-restricted feeding (TRF) is a mode of intermittent fasting in which the daily eating window is restricted to 4–12 h [11]. Recent studies have indicated that TRF improves insulin sensitivity [12], 24 h glucose levels [6], glucose tolerance [13], lipid metabolism, the circadian clock [6,14], autophagy [6], and cardiac dysfunction [15], as well as metabolic dysfunction-associated steatotic liver disease (MASLD) [16]. In particular, the EASL-EASD-EASO Clinical Practice Guidelines on the management of metabolic dysfunction-associated steatotic liver disease (MASLD) detail the effect of TRF on MASLD [17,18]. Another network meta-analysis of 59 randomized controlled trials in subjects with MASLD showed that TRF certainly improves short-term outcomes including mortality, liver-related complications, and hepatic cancer [17,19]. Collectively, TRF can be considered a potential countermeasure for the prevention and treatment of obesity and metabolic disorders and the restoration of circadian rhythm; it may also have anti-aging effects in humans [20,21]. While several studies demonstrate that TRF is effective for weight loss [22,23], the role of TRF in weight loss remains controversial [24,25]. TRF has several benefits for the metabolism; however, the impact that TRF has on the action of the neuroendocrine system is still unknown. LEAP-2 is a novel antagonist against ghrelin and is involved in the orexigenic system. There are no data currently available regarding the association of LEAP-2 with the amount of food consumed in the TRF state. The purpose of this research is to investigate how central administration of LEAP-2 influences eating behavior, which is evaluated according to cumulative food intake in the TRF state in rats.

## 2. Materials and Methods

### 2.1. Animals

A total of 72 male Sprague Dawley rats weighing 110–140 g (being three weeks old) were purchased from BioLASCO Taiwan Co., Ltd. (Taipei, Taiwan) and kept in the Laboratory Animal Center, National Yang Ming Chiao Tung University (Taipei, Taiwan). The rats were kept in rooms with controlled illumination (light period: 08:00–20:00), humidity (60 ± 10%), and temperature (22.5 ± 1.5 °C). All rats had free water and laboratory chow pellets (BioLASCO Taiwan Co., Ltd., Taipei, Taiwan). All animal protocols were conducted after 8:00 AM in conscious rats, according to the guidelines regulated by the Institutional Animal Care and Use Committee (IACUC) of the Taipei Veterans General Hospital, Taiwan (approved protocol number: IACUC 2022-029). The 72 rats were divided into 6 groups, including vehicle + vehicle, vehicle + LEAP-2, ghrelin + vehicle, and ghrelin + LEAP-2 (0.1, 1, 3 nmol/rat). Based on ethology experiments and previous studies [10,26,27,28], 12 rats were included in each group.

### 2.2. Implantation of an Intracerebroventricular (ICV) Catheter

Before administration of vehicle or *O-n*-octanoylated ghrelin (0.1 nmol/rat) via ICV, we administered vehicle or LEAP-2 (0.1 nmol/rat, 1 nmol/rat, or 3 nmol/rat) via ICV. Before ICV administration, all rats were anesthetized with intraperitoneal (IP) injection of Zoletil-50 (20–40 mg/kg, Tiletamine: Zolazepam = 1:1; Virbac Taiwan Co. Ltd. (Kaohsiung, Taiwan)) and Xylazine (1–5 mg/kg, Rompun, BioLASCO Taiwan Co., Ltd. (Taipei, Taiwan)) [10,26,27,28]. Then, all rats were placed in a stereotaxic apparatus (BenchmarkTM, myNeuroLab, St. Louis, MO, USA) and received an implant of a guide cannula (25-gauge; Eicom, Kyoto, Japan), which reached the right lateral ventricle. The coordinates of the stereotaxic apparatus were 0.8 mm posterior to bregma, 1.4 mm right lateral to the midline, and 4.5 mm below the outer surface of the skull using a stereotaxic frame with the incisor bar set within the horizontal plane passing through the bregma and lambda [10,28,29]. Securing of the guide cannula, insertion of a dummy cannula (Eicom, Kyoto, Japan) into the guide cannula, and placement of a screw cap (Eicom, Kyoto, Japan) were performed as described in our previous studies [10,28,29]. The rats were isolated and given at least 7 days for full recovery after the implantation of ICV catheters before the food intake study. All ICV injections lasted for 60 s with the total volume of 10 μL via AMI-5 (Eicom, Kyoto, Japan).

### 2.3. Preparation of Drugs

Liver-expressed antimicrobial peptide-2 (LEAP-2, Abbexa Ltd., Cambridge, UK) and rat *O-n*-octanoylated ghrelin (American Peptide Co., Sunnyvale, CA, USA) were kept in powder form at –20 °C and dissolved in sterile, pyrogen-free 0.9% saline (Otsuka, Tokyo, Japan) immediately before use.

### 2.4. Food Intake Analysis

The measurement of food intake was performed as in our previous studies with a slight modification [28]. Briefly, the cumulative food intake was recorded and calculated at 1, 2, 4, 8, 12, and 24 h after ICV injection. The total volume of ICV injection was 10 μL for each rat. Food intake was determined by measuring the difference between the pre-weighed standard chow and weight of chow at each time point. Before the food intake tests, all rats were allowed to fully acclimatize to their environments for one week. Light-phase experiments on either freely fed or TRF rats were started at 8:00 a.m. Before the tests, the rats were given free access to food and water (i.e., freely fed). A standard diet (BioLASCO Taiwan Co., Ltd., Taipei, Taiwan) was provided.

### 2.5. Experimental Feeding Schedule: Ad Libitum (AL) versus Time-Restricted Feeding (TRF)

Although LEAP-2 is an endogenous antagonist of the ghrelin receptor [1], central LEAP-2 has been shown to suppress only central ghrelin-induced feeding, while failing to inhibit either 14 h fasting-induced or dark-phase food intake in rats [6]. These data highlight the more pivotal role of LEAP-2 in the fed state rather than caloric restriction in the regulation of ingestive behavior [1,6]. Therefore, we designed our food intake analysis to be performed in a freely fed satiated state instead of a food-deprived state.

In addition to the control group having AL access to food, an animal model for TRF was also established. Rats under the TRF protocol were allowed access to the diet for 8 h per day during the active phase (dark period) from ZT 13 (1 h after lights on) to ZT 21 (3 h before lights on) for 28 days [22]. Rats fed either AL or TRF were transferred between feeding cages at the same time in order to standardize handling stress and minimize experimental variation between groups. In our previous serial published papers [26,27,28,30], we have already created a stable and reproducible model of ICV acyl ghrelin-induced feeding in freely fed rats. We applied this ICV acyl ghrelin-induced feeding model in either AL or TRF rats.

### 2.6. Statistical Analyses

All results are expressed as mean ± SEM, and SigmaStat 4.0 software (Grafiti LLC., Palo Alto, CA, USA) was used for data analysis. A one-way ANOVA was conducted to compare the difference among groups, and the Student–Newman–Keuls post hoc test was conducted to identify the difference between each pair of groups. Statistical significance was defined at *p* < 0.05.

## 3. Results

### 3.1. Action of Pre-Treatment with LEAP-2 via ICV without O-n-Octanoylated Ghrelin

**Figure 1:**
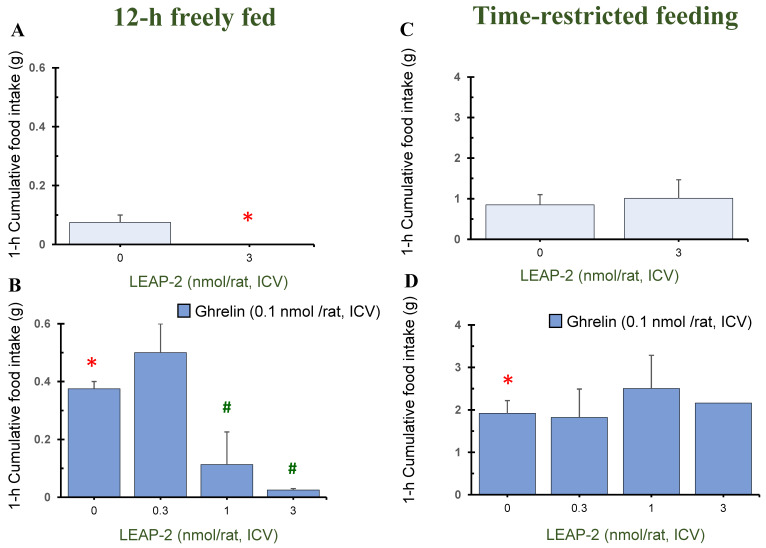
Cumulative food intake after 1 h in rats in 12 h freely fed and time-restricted feeding states. Cumulative food intake in rats was measured over a period of 1 h. Cumulative food intake after 1 h in rats in 12 h freely fed (**A**,**B**) and time-restricted feeding states (**C**,**D**) in the light phase. (**A**) Cumulative food intake after 1 h in rats with or without LEAP-2 (3 nmol/rat, ICV) in a freely fed state, where both groups did not receive *O-n*-octanoylated ghrelin. (**B**) Cumulative food intake after 1 h in rats with vehicle or LEAP-2 (0.3, 1, 3 nmol/rat, ICV) before receiving *O-n*-octanoylated ghrelin (0.1 nmol/rat) in a 12 h freely fed state. (**C**) Cumulative food intake after 1 h in rats with or without LEAP-2 (3 nmol/rat, ICV) in a time-restricted feeding state, where both groups did not receive *O-n*-octanoylated ghrelin. (**D**) Cumulative food intake after 1 h in rats with vehicle or LEAP-2 (0.3, 1, 3 nmol/rat, ICV) before receiving *O-n*-octanoylated ghrelin (0.1 nmol/rat) in a time-restricted feeding state. The sample size for each experimental group in each feeding state was 12 rats. * *p* < 0.05 vs. vehicle + vehicle in each feeding state. # *p* < 0.05 vs. vehicle + *O-n*-octanoylated ghrelin in each feeding state. LEAP-2: liver-expressed antimicrobial peptide-2; ICV: intracerebroventricular injection.

**Figure 2:**
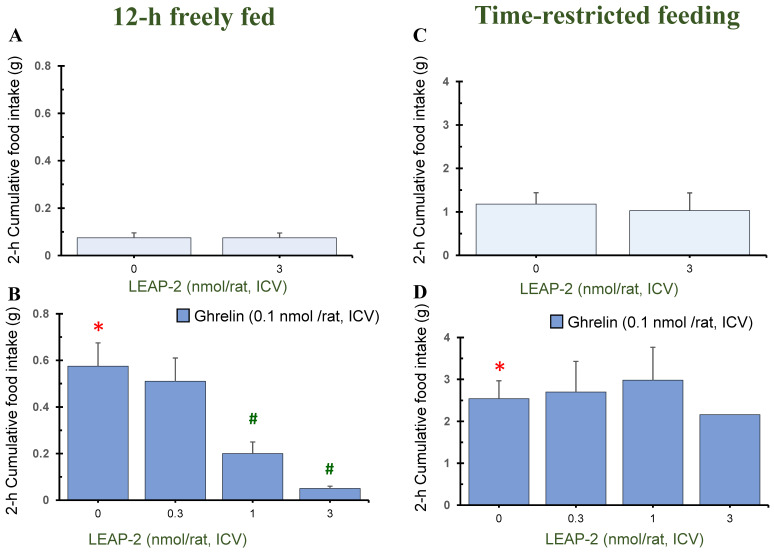
Cumulative food intake after 2 h in rats in 12 h freely fed and time-restricted feeding states. Cumulative food intake in rats was measured over a period of 2 h. Cumulative food intake after 2 h in rats in 12 h freely fed (**A**,**B**) and time-restricted feeding states (**C**,**D**) in the light phase. (**A**) Cumulative food intake after 2 h in rats with or without LEAP-2 (3 nmol/rat, ICV) in a freely fed state, where both groups did not receive *O-n*-octanoylated ghrelin. (**B**) Cumulative food intake after 2 h in rats with vehicle or LEAP-2 (0.3, 1, 3 nmol/rat, ICV) before receiving *O-n*-octanoylated ghrelin (0.1 nmol/rat) in a 12 h freely fed state. (**C**) Cumulative food intake after 2 h in rats with or without LEAP-2 (3 nmol/rat, ICV) in a time-restricted feeding state, where both groups did not receive *O-n*-octanoylated ghrelin. (**D**) Cumulative food intake after 2 h in rats with vehicle or LEAP-2 (0.3, 1, 3 nmol/rat, ICV) before receiving *O-n*-octanoylated ghrelin (0.1 nmol/rat) in a time-restricted feeding state. The sample size for each experimental group in each feeding state was 12 rats. * *p* < 0.05 vs. vehicle + vehicle in each feeding state. # *p* < 0.05 vs. vehicle + *O-n*-octanoylated ghrelin in each feeding state. LEAP-2: liver-expressed antimicrobial peptide-2; ICV: intracerebroventricular injection.

**Figure 3:**
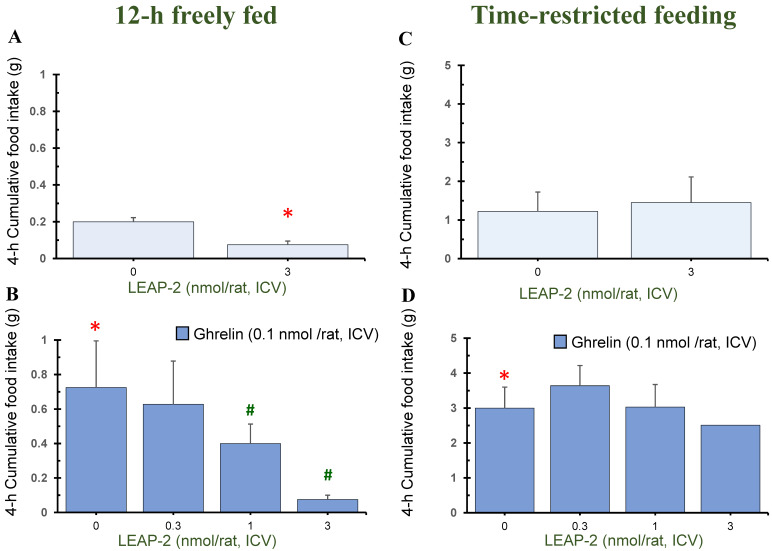
Cumulative food intake after 4 h in rats in 12 h freely fed and time-restricted feeding states. Cumulative food intake in rats was measured over a period of 4 h. Cumulative food intake after 4 h in rats in 12 h freely fed (**A**,**B**) and time-restricted feeding states (**C**,**D**) in the light phase. (**A**) Cumulative food intake after 4 h in rats with or without LEAP-2 (3 nmol/rat, ICV) in a freely fed state, where both groups did not receive *O-n*-octanoylated ghrelin. (**B**) Cumulative food intake after 4 h in rats with vehicle or LEAP-2 (0.3, 1, 3 nmol/rat, ICV) before receiving *O-n*-octanoylated ghrelin (0.1 nmol/rat) in a 12 h freely fed state. (**C**) Cumulative food intake after 4 h in rats with or without LEAP-2 (3 nmol/rat, ICV) in a time-restricted feeding state, where both groups did not receive *O-n*-octanoylated ghrelin. (**D**) Cumulative food intake after 4 h in rats with vehicle or LEAP-2 (0.3, 1, 3 nmol/rat, ICV) before receiving *O-n*-octanoylated ghrelin (0.1 nmol/rat) in a time-restricted feeding state. The sample size for each experimental group in each feeding state was 12 rats. * *p* < 0.05 vs. vehicle + vehicle in each feeding state. # *p* < 0.05 vs. vehicle + *O-n*-octanoylated ghrelin in each feeding state. LEAP-2: liver-expressed antimicrobial peptide-2; ICV: intracerebroventricular injection.

**Figure 4:**
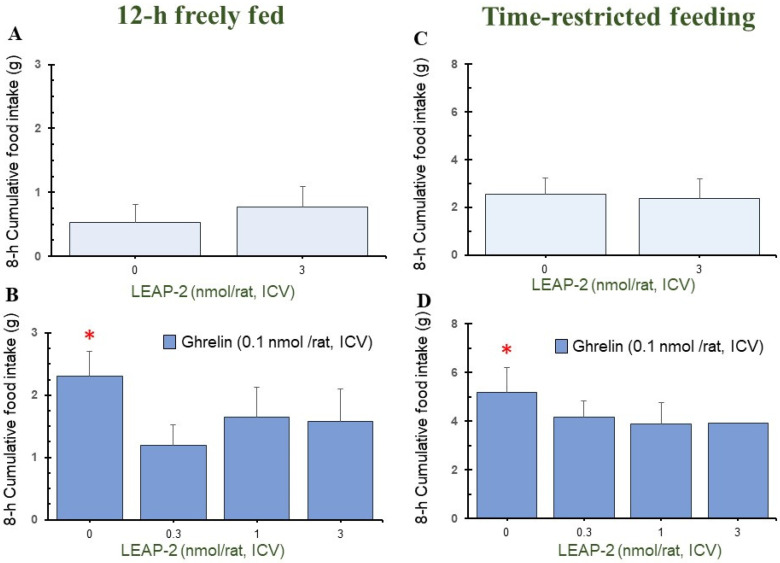
Cumulative food intake after 8 h in rats in 12 h freely fed and time-restricted feeding states. Cumulative food intake in rats was measured over a period of 8 h. Cumulative food intake after 8 h in rats in 12 h freely fed (**A**,**B**) and time-restricted feeding states (**C**,**D**) in the light phase. (**A**) Cumulative food intake after 8 h in rats with or without LEAP-2 (3 nmol/rat, ICV) in a freely fed state, where both groups did not receive *O-n*-octanoylated ghrelin. (**B**) Cumulative food intake after 8 h in rats with vehicle or LEAP-2 (0.3, 1, 3 nmol/rat, ICV) before receiving *O-n*-octanoylated ghrelin (0.1 nmol/rat) in a 12 h freely fed state. (**C**) Cumulative food intake after 8 h in rats with or without LEAP-2 (3 nmol/rat, ICV) in a time-restricted feeding state, where both groups did not receive *O-n*-octanoylated ghrelin. (**D**) Cumulative food intake after 8 h in rats with vehicle or LEAP-2 (0.3, 1, 3 nmol/rat, ICV) before receiving *O-n*-octanoylated ghrelin (0.1 nmol/rat) in a time-restricted feeding state. The sample size for each experimental group in each feeding state was 12 rats. * *p* < 0.05 vs. vehicle + vehicle in each feeding state. LEAP-2: liver-expressed antimicrobial peptide-2; ICV: intracerebroventricular injection.

**Figure 5:**
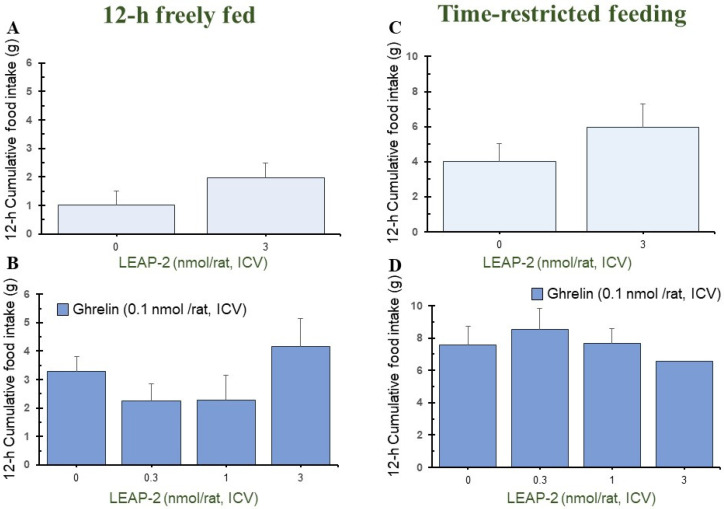
Cumulative food intake after 12 h in rats in 12 h freely fed and time-restricted feeding states. Cumulative food intake in rats was measured over a period of 12 h. Cumulative food intake after 12 h in rats in 12 h freely fed (**A**,**B**) and time-restricted feeding states (**C**,**D**) in the light phase. (**A**) Cumulative food intake after 12 h in rats with or without LEAP-2 (3 nmol/rat, ICV) in a freely fed state, where both groups did not receive *O-n*-octanoylated ghrelin. (**B**) Cumulative food intake after 12 h in rats with vehicle or LEAP-2 (0.3, 1, 3 nmol/rat, ICV) before receiving *O-n*-octanoylated ghrelin (0.1 nmol/rat) in a 12 h freely fed state. (**C**) Cumulative food intake after 12 h in rats with or without LEAP-2 (3 nmol/rat, ICV) in a time-restricted feeding state, where both groups did not receive *O-n*-octanoylated ghrelin. (**D**) Cumulative food intake after 12 h in rats with vehicle or LEAP-2 (0.3, 1, 3 nmol/rat, ICV) before receiving *O-n*-octanoylated ghrelin (0.1 nmol/rat) in a time-restricted feeding state. The sample size for each experimental group in each feeding state was 12 rats. LEAP-2: liver-expressed antimicrobial peptide-2; ICV: intracerebroventricular injection.

**Figure 6:**
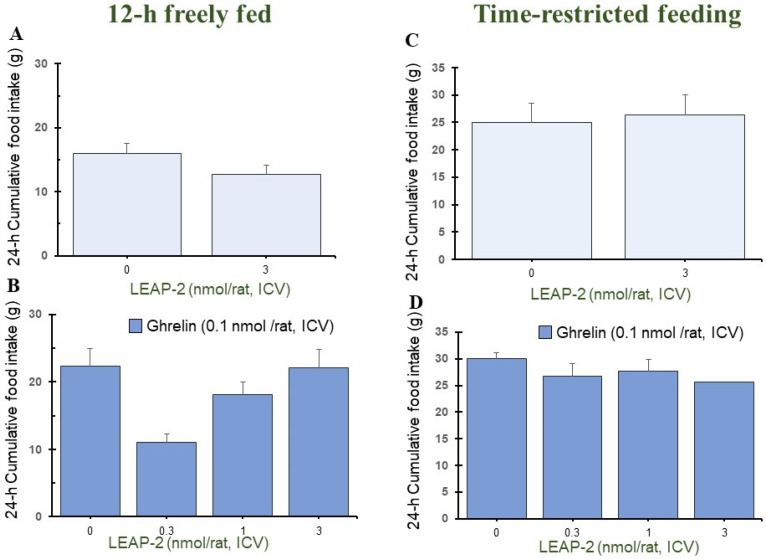
Cumulative food intake after 24 h in rats in 12 h freely fed and time-restricted feeding states. Cumulative food intake in rats was measured over a period of 24 h. Cumulative food intake after 24 h in rats in 12 h freely fed (**A**,**B**) and time-restricted feeding states (**C**,**D**) in the light phase. (**A**) Cumulative food intake after 24 h in rats with or without LEAP-2 (3 nmol/rat, ICV) in a freely fed state, where both groups did not receive *O-n*-octanoylated ghrelin. (**B**) Cumulative food intake after 24 h in rats with vehicle or LEAP-2 (0.3, 1, 3 nmol/rat, ICV) before receiving *O-n*-octanoylated ghrelin (0.1 nmol/rat) in a 12 h freely fed state. (**C**) Cumulative food intake after 24 h in rats with or without LEAP-2 (3 nmol/rat, ICV) in a time-restricted feeding state, where both groups did not receive *O-n*-octanoylated ghrelin. (**D**) Cumulative food intake after 24 h in rats with vehicle or LEAP-2 (0.3, 1, 3 nmol/rat, ICV) before receiving *O-n*-octanoylated ghrelin (0.1 nmol/rat) in a time-restricted feeding state. The sample size for each experimental group in each feeding state was 12 rats. LEAP-2: liver-expressed antimicrobial peptide-2; ICV: intracerebroventricular injection.

Pre-treatment with LEAP-2 (3 nmol/rat) via ICV without *O-n*-octanoylated ghrelin lowered the cumulative food intake in rats at 1 h, 4 h, and 8 h in a 12 h freely fed state, compared with rats without pre-treatment with LEAP-2 (Figures 1A, 3A and 4A). There was no significant difference at 2 h, 12 h, and 24 h (Figures 2A, 5A and 6A).

In the TRF state, there was no significant difference in cumulative food intake between vehicle + vehicle and vehicle + LEAP-2 groups from 1 h to 24 h (Figure 1C, Figure 2C, Figure 3C, Figure 4C, Figure 5C and Figure 6C).

### 3.2. Effect of Interaction between LEAP-2 and O-n-Octanoylated Ghrelin on Food Intake in a 12 h Freely Fed State

Before administration of *O-n*-octanoylated ghrelin (0.1 nmol/rat), pre-treatment with LEAP-2 inhibited cumulative food intake in the 12 h freely fed state at 1 h (Figure 1B), 2 h (Figure 2B), and 4 h (Figure 3B), which indicates that the hyperphagic action of ghrelin was blocked by LEAP-2. Moreover, both 1 and 3 nmol/rat LEAP-2 ICV administration significantly decreased 1 h (Figure 1B), 2 h (Figure 2B) and 4 h (Figure 3B) cumulative food intake, compared with administration of 0.3 nmol/rat LEAP-2. There was no significant difference in cumulative food intake among the LEAP-2 (0.3 nmol, ICV) + ghrelin (0.1 nmol/rat, ICV), LEAP-2 (1 nmol, ICV) + ghrelin (0.1 nmol/rat, ICV), and LEAP-2 (3 nmol, ICV) + ghrelin (0.1 nmol/rat, ICV) groups from 8 to 24 h (Figure 4B, Figure 5B and Figure 6B).

### 3.3. Effect of Interaction between LEAP-2 and O-n-Octanoylated Ghrelin on Food Intake under TRF State

Before the administration of *O-n*-octanoylated ghrelin via ICV, the doses (0.3, 1, and 3 nmol/rat) of LEAP-2 did not inhibit cumulative food intake in the TRF state (Figure 1D, Figure 2D, Figure 3D, Figure 4D, Figure 5D and Figure 6D) within 24 h. There was no significant difference in the cumulative food intake among vehicle + ghrelin (0.1 nmol/rat), LEAP-2 (0.3 nmol, ICV) + ghrelin (0.1 nmol/rat, ICV), LEAP-2 (1 nmol/rat, ICV) + ghrelin (0.1 nmol/rat, ICV), and LEAP-2 (3 nmol/rat, ICV) + ghrelin (0.1 nmol/rat, ICV) groups from 1 to 24 h (Figure 1D, Figure 2D, Figure 3D, Figure 4D, Figure 5D and Figure 6D).

### 3.4. Temporal Effects of Pre-Treatment ICV Administration of LEAP-2

**Figure 7:**
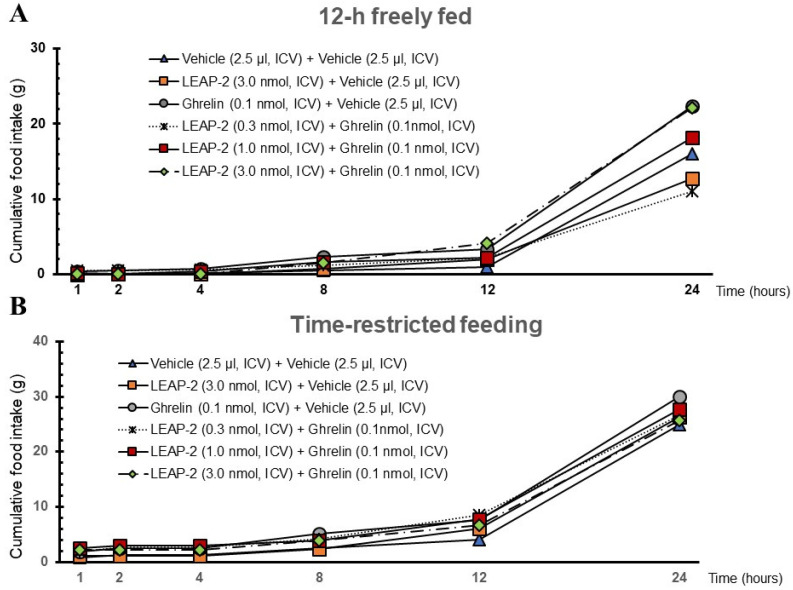
Temporal relationships between LEAP-2, *O-n*-octanoylated ghrelin, and cumulative food intake in 12 h freely fed and time-restricted states. (**A**) Cumulative food intake at 1 h, 2 h, 4 h, 8 h, 12 h, and 24 h in a 12 h freely fed state. (**B**) Cumulative food intake at 1 h, 2 h, 4 h, 8 h, 12 h, and 24 h in a time-restricted feeding state. The sample size for each experimental group in each feeding state was 12 rats. LEAP-2: liver-expressed antimicrobial peptide-2; ICV: intracerebroventricular injection.

Cumulative food intake was assessed over a 24 h period, during which rats were in a 12 h freely fed state following ICV administration of different treatments. The rats treated with LEAP-2 showed a decreased appetite, as evidenced by lower food intake, while *O-n*-octanoylated ghrelin administration appeared to increase food consumption. Interestingly, the co-administration of LEAP-2 and ghrelin resulted in a food intake pattern that suggested a mitigating effect of LEAP-2 on the appetite-stimulating properties of ghrelin (Figure 7A). Despite administration of ghrelin and LEAP-2, there was no significant difference in cumulative food intake from 1 to 24 h in the TRF state (Figure 7B).

### 3.5. Cumulative Food Intake after Administration of LEAP-2 with or without Ghrelin

In the 12 h freely fed state, the cumulative food intake of rats administered *O-n*-octanoylated ghrelin (0.1 nmol/rat) was higher compared to those rats receiving vehicle in the absence of LEAP-2 (*p* < 0.05). However, pre-treatment with LEAP-2 (3 nmol/rat, ICV) before ghrelin administration significantly decreased cumulative food intake compared with rats only receiving *O-n*-octanoylated ghrelin (*p* < 0.05). Without ghrelin administration, LEAP-2 alone also significantly reduced cumulative food intake compared to the vehicle group (*p* < 0.05). Interestingly, the cumulative food intake of rats receiving ghrelin in the time-restricted feeding (TRF) state was significantly increased compared to those in the 12 h freely fed state with ghrelin. The various effects mentioned above were sustained from 1 to 4 h (Supplementary Materials).

## 4. Discussion

In the present study, the administration of *O-n*-octanoyl ghrelin through ICV increased food intake in both the freely fed and TRF groups. This result is consistent with other research conducted by scholars [27,31,32,33,34]. Furthermore, previous studies have demonstrated that the administration of ghrelin via intravenous injection at a dosage of 5.0 pmol/kg/min had significant effects on the enhancement of appetite and food consumption in human participants [35,36]. In humans, ghrelin is a hormone that stimulates appetite and is found in two different forms: *O-n*-octanoyl ghrelin and des-acyl ghrelin [37,38,39]. The enzymatic process of *O*-acylation with octanoate, assisted by the enzyme ghrelin *O*-acyltransferase (GOAT), is responsible for the conversion of des-acyl ghrelin on serine-3 to *O-n*-octanoyl ghrelin [40]. The primary orexigenic mechanism through which ghrelin stimulates feeding is activation of the growth hormone secretagogue receptor in the hypothalamus. Neuropeptide Y and agouti-related proteins are activated more strongly in the arcuate nucleus (ARC) [33]. Subsequently, the orexin neurons in the laterodorsal tegmental nucleus are activated, resulting in the stimulation of appetite and a subsequent increase in food consumption [33].

In the present study, the administration of *O-n*-octanoyl ghrelin through ICV increased food intake in both the freely fed and TRF groups. This result is consistent with previous research. The ICV injection of *O-n*-octanoyl ghrelin, when administered with LEAP-2, successfully inhibited activity of ghrelin, as evaluated through quantifying food consumption during the 12 h freely fed period, a result which is in agreement with prior research [1,6]. In a separate investigation, mice were injected with LEAP-2 at a concentration of 3 μmol/kg body weight through a subcutaneous route, followed by the intravenous administration of acyl ghrelin at a dosage of 0.15 μmol/kg body weight [1]. The administration of LEAP-2 was shown to result in a decrease in the total food consumption of mice within a time frame of 2 h, as compared to animals that did not receive LEAP-2 [1]. The inhibition of ghrelin-induced food intake through peripheral injection of LEAP-2 was observed only when the dosage of LEAP-2 exceeded the dosage of peripheral ghrelin by three times [1,4,6]. Furthermore, current research has suggested that administering LEAP-2 as a central treatment leads to a significant reduction in food consumption [6,41]. Cornejo et al. reported that injection of LEAP-2 (600 pmol/mouse) through the intracerebral vein led to a decrease in the cumulative high-fat intake of mice over a period of four days [41]. In another study, pre-treatment with LEAP-2 (10 pmol/mouse) via ICV administration before ICV administration of ghrelin (60 pmol/mouse) also resulted in a 40% decrease in food intake at the second hour [42]. Importantly, the current study confirmed that pre-treatment with LEAP-2 (1 nmol/rat and 3 nmol/rat) administered through ICV not only inhibited the appetite-inducing effects of ghrelin (0.1 nmol/rat) during a 12 h period of unrestricted feeding but also demonstrated that the appetite-suppressing action of LEAP-2 is dose-related.

Several studies have indicated that LEAP-2 inhibits the activity of ghrelin-induced orexigenic effects [1,5,6,41]. Despite the administration of LEAP-2 via ICV, the cumulative food intake of the rats was still high, as those rats received vehicle under TRF in the current study. The plausible mechanisms of pre-treatment with LEAP-2 not suppressing the activities of ghrelin in the TRF state might be related to (1) the mutual antagonism between ghrelin and LEAP-2; (2) the LEAP-2/ghrelin molar ratio; (3) the fact that LEAP-2 does not inhibit neuropeptide Y-induced food intake, which suggests that the inhibitory effects of LEAP-2 are specific to GHSR [6]; and (4) the elimination half-life of gut–liver hormone. Ghrelin and LEAP-2 engaged in mutual antagonism and remained in balance in the subjects [43,44,45]. LEAP-2 inhibits ghrelin through its antagonism of GHSR, as mentioned above, and ghrelin suppresses the expression of LEAP-2 through activating a GHS-R1a-AMP-activated protein kinase (AMPK)-dependent pathway [6]. Additionally, LEAP-2 inhibited food ingestion during satiated state, when the LEAP-2/ghrelin molar ratio was about 10–20, which might be related to the high constitutive activity of GHS-R1a [1,5,45]. Hence, a higher LEAP-2/ghrelin molar ratio might be necessary for LEAP-2 to suppress the activity of ghrelin. Moreover, another reason might be that TRF could elevate the level of endogenous ghrelin, which remains unaffected by LEAP-2 inhibition during TRF. Previous research has indicated that the endogenous ghrelin level rises in the TRF state and during the early stages of diabetes mellitus, obesity, and anorexia–cachexia [7,9,46,47]. Another plausible reason that LEAP-2 did not inhibit ghrelin might be related to the different elimination half-lives of ghrelin and LEAP-2; in particular, the elimination half-life of ghrelin (27–31 min) is longer than that of LEAP-2 (about 9 min) [48,49]. LEAP-2 is an unstable peptide that is rapidly proteolyzed into fragmental peptides [48,50]. Furthermore, the effectiveness of TRF in improving metabolic disease is still contentious. The study period of most studies on TRF in metabolic diseases (e.g., obesity and MASLD) is less than three months [51,52,53], while the long-term outcomes of TRF are still unknown [17]. Moreover, most studies have demonstrated that the effectiveness of TRF on MASLD should be combined with exercise [52]. Sutton et al. demonstrated that TRF truly attenuates blood pressure and oxidative stress, rather than food intake and weight loss, in humans over a 5-week study period [51]. The reason that weight loss fails might be related to the type of intermittent fasting [51], meal timing [54], caloric intake restriction, and combination of exercise [52]. The results from our study might provide neuroendocrine-based evidence to explain why TRF sometimes fails in fighting obesity and/or MASLD in clinical practice. Finally, further investigation is warranted to elucidate the detailed mechanism of LEAP-2 in the TRF state.

## 5. Conclusions

Centrally administered LEAP-2 truly inhibits *O-n*-octanoylated ghrelin-induced eating behaviors in dose-dependent manner in a freely fed but not a TRF state. TRF is widely applied for weight loss and improvement of metabolic disease (e.g., obesity and MASLD). It was found that central administration of LEAP-2 could not suppress *O-n*-octanoylated ghrelin-induced food intake in the TRF state. This study may provide explanations as to why TRF fails to improve metabolic disease and induce changes in ingestion behavior, one of which may be disruption of the balance between ghrelin and LEAP-2.

## Data Availability

The datasets are available from the corresponding author at chency@vghtpe.gov.tw.

## Supplementary Materials

The following supporting information can be downloaded at: https://www.mdpi.com/article/10.3390/nu16121946/s1, Figure S1. Comparison of 1 h cumulative food intake in 12 h freely fed and time-restricted feeding states. The rats were administered with vehicle, *O-n*-octanoylated ghrelin (0.1 nmol/rat, ICV), LEAP-2 (3 nmol/rat, ICV), and *O-n*-octanoylated ghrelin (0.1 nmol/rat, ICV) + pre-treatment with LEAP-2 (3 nmol/rat, ICV). The sample size for each experimental group in each feeding state was 12 rats. LEAP-2: liver-expressed antimicrobial peptide-2; ICV: intracerebroventricular injection. * *p* < 0.05 vs. vehicle + vehicle, # *p* < 0.05 vs. vehicle + ghrelin. Figure S2. Comparison of 2 h cumulative food intake in 12 h freely fed and time-restricted feeding states. The rats were administered with vehicle, *O-n*-octanoylated ghrelin (0.1 nmol/rat, ICV), LEAP-2 (3 nmol/rat, ICV), and *O-n*-octanoylated ghrelin (0.1 nmol/rat, ICV) + pre-treatment with LEAP-2 (3 nmol/rat, ICV). The sample size for each experimental group in each feeding state was 12 rats. LEAP-2: liver-expressed antimicrobial peptide-2; ICV: intracerebroventricular injection. * *p* < 0.05 vs. vehicle + vehicle, # *p* < 0.05 vs. vehicle + ghrelin. Figure S3. Comparison of 4 h cumulative food intake in 12 h freely fed and time-restricted feeding states. The rats were administered with vehicle, *O-n*-octanoylated ghrelin (0.1 nmol/rat, ICV), LEAP-2 (3 nmol/rat, ICV), and *O-n*-octanoylated ghrelin (0.1 nmol/rat, ICV) + pre-treatment with LEAP-2 (3 nmol/rat, ICV). The sample size for each experimental group in each feeding state was 12 rats. LEAP-2: liver-expressed antimicrobial peptide-2; ICV: intracerebroventricular injection. * *p* < 0.05 vs. vehicle + vehicle, # *p* < 0.05 vs. vehicle + ghrelin.

## References

[1] Ge X., Yang H., Bednarek M.A., Galon-Tilleman H., Chen P., Chen M., Lichtman J.S., Wang Y., Dalmas O., Yin Y. (2018). LEAP2 Is an Endogenous Antagonist of the Ghrelin Receptor. Cell Metab..

[2] Lugilde J., Casado S., Beiroa D., Cuñarro J., Garcia-Lavandeira M., Álvarez C.V., Nogueiras R., Diéguez C., Tovar S. (2022). LEAP-2 Counteracts Ghrelin-Induced Food Intake in a Nutrient, Growth Hormone and Age Independent Manner. Cells.

[3] Ma X., Xue X., Zhang J., Liang S., Xu C., Wang Y., Zhu J. (2021). Liver Expressed Antimicrobial Peptide 2 is Associated with Steatosis in Mice and Humans. Exp. Clin. Endocrinol. Diabetes.

[4] M’Kadmi C., Cabral A., Barrile F., Giribaldi J., Cantel S., Damian M., Mary S., Denoyelle S., Dutertre S., Péraldi-Roux S. (2019). N-Terminal Liver-Expressed Antimicrobial Peptide 2 (LEAP2) Region Exhibits Inverse Agonist Activity toward the Ghrelin Receptor. J. Med. Chem..

[5] Lu X., Huang L., Huang Z., Feng D., Clark R.J., Chen C. (2021). LEAP-2: An Emerging Endogenous Ghrelin Receptor Antagonist in the Pathophysiology of Obesity. Front. Endocrinol..

[6] Islam M.N., Mita Y., Maruyama K., Tanida R., Zhang W., Sakoda H., Nakazato M. (2020). Liver-expressed antimicrobial peptide 2 antagonizes the effect of ghrelin in rodents. J. Endocrinol..

[7] Chen C.Y., Asakawa A., Fujimiya M., Lee S.D., Inui A. (2009). Ghrelin gene products and the regulation of food intake and gut motility. Pharmacol. Rev..

[8] Mizutani M., Atsuchi K., Asakawa A., Matsuda N., Fujimura M., Inui A., Kato I., Fujimiya M. (2009). Localization of acyl ghrelin- and des-acyl ghrelin-immunoreactive cells in the rat stomach and their responses to intragastric pH. Am. J. Physiol.-Gastrointest. Liver Physiol..

[9] Chen C.Y., Fujimiya M., Laviano A., Chang F.Y., Lin H.C., Lee S.D. (2010). Modulation of ingestive behavior and gastrointestinal motility by ghrelin in diabetic animals and humans. J. Chin. Med. Assoc..

[10] Chen C.Y., Inui A., Asakawa A., Fujino K., Kato I., Chen C.C., Ueno N., Fujimiya M. (2005). Des-acyl ghrelin acts by CRF type 2 receptors to disrupt fasted stomach motility in conscious rats. Gastroenterology.

[11] Varady K.A. (2011). Intermittent versus daily calorie restriction: Which diet regimen is more effective for weight loss?. Obes. Rev..

[12] Das M., Kumar D., Sauceda C., Oberg A., Ellies L.G., Zeng L., Jih L.J., Newton I.G., Webster N.J.G. (2024). Time-Restricted Feeding Attenuates Metabolic Dysfunction-Associated Steatohepatitis and Hepatocellular Carcinoma in Obese Male Mice. Cancers.

[13] Hutchison A.T., Regmi P., Manoogian E.N.C., Fleischer J.G., Wittert G.A., Panda S., Heilbronn L.K. (2019). Time-Restricted Feeding Improves Glucose Tolerance in Men at Risk for Type 2 Diabetes: A Randomized Crossover Trial. Obesity.

[14] Zeb F., Wu X., Fatima S., Zaman M.H., Khan S.A., Safdar M., Alam I., Feng Q. (2021). Time-restricted feeding regulates molecular mechanisms with involvement of circadian rhythm to prevent metabolic diseases. Nutrition.

[15] Wang X.P., Xing C.Y., Zhang J.X., Zhou J.H., Li Y.C., Yang H.Y., Zhang P.F., Zhang W., Huang Y., Long J.G. (2020). Time-restricted feeding alleviates cardiac dysfunction induced by simulated microgravity via restoring cardiac FGF21 signaling. FASEB J..

[16] Zelber-Sagi S., Grinshpan L.S., Ivancovsky-Wajcman D., Goldenshluger A., Gepner Y. (2022). One size does not fit all; practical, personal tailoring of the diet to NAFLD patients. Liver Int..

[17] Tacke F., Horn P., Wai-Sun Wong V., Ratziu V., Bugianesi E., Francque S., Zelber-Sagi S., Valenti L., Roden M., Schick F. (2024). EASL-EASD-EASO Clinical Practice Guidelines on the management of metabolic dysfunction-associated steatotic liver disease (MASLD). Obes. Facts.

[18] Zelber-Sagi S. (2024). Guidelines and Future Perspectives of MAFLD. Obes. Facts.

[19] Buzzetti E., Linden A., Best L.M., Madden A.M., Roberts D., Chase T.J.G., Freeman S.C., Cooper N.J., Sutton A.J., Fritche D. (2021). Lifestyle modifications for nonalcohol-related fatty liver disease: A network meta-analysis. Cochrane Database Syst. Rev..

[20] Xie Z., Sun Y., Ye Y., Hu D., Zhang H., He Z., Zhao H., Yang H., Mao Y. (2022). Randomized controlled trial for time-restricted eating in healthy volunteers without obesity. Nat. Commun..

[21] Manoogian E.N.C., Panda S. (2017). Circadian rhythms, time-restricted feeding, and healthy aging. Ageing Res. Rev..

[22] Jamshed H., Steger F.L., Bryan D.R., Richman J.S., Warriner A.H., Hanick C.J., Martin C.K., Salvy S.-J., Peterson C.M. (2022). Effectiveness of Early Time-Restricted Eating for Weight Loss, Fat Loss, and Cardiometabolic Health in Adults With Obesity: A Randomized Clinical Trial. JAMA Intern. Med..

[23] Liu X., Xu Y., Mu X., Shen J. (2024). The effects of time restricted feeding on weight loss and other changes of anthropometric parameters among physically active individuals. Sci. Sports.

[24] Phillips N.E., Mareschal J., Schwab N., Manoogian E.N.C., Borloz S., Ostinelli G., Gauthier-Jaques A., Umwali S., Gonzalez Rodriguez E., Aeberli D. (2021). The Effects of Time-Restricted Eating versus Standard Dietary Advice on Weight, Metabolic Health and the Consumption of Processed Food: A Pragmatic Randomised Controlled Trial in Community-Based Adults. Nutrients.

[25] Lowe D.A., Wu N., Rohdin-Bibby L., Moore A.H., Kelly N., Liu Y.E., Philip E., Vittinghoff E., Heymsfield S.B., Olgin J.E. (2020). Effects of Time-Restricted Eating on Weight Loss and Other Metabolic Parameters in Women and Men With Overweight and Obesity: The TREAT Randomized Clinical Trial. JAMA Intern. Med..

[26] Ting C.H., Chi C.W., Li C.P., Chen C.Y. (2015). Differential modulation of endogenous cannabinoid CB1 and CB2 receptors in spontaneous and splice variants of ghrelin-induced food intake in conscious rats. Nutrition.

[27] Yeh C., Ting C.H., Doong M.L., Chi C.W., Lee S.D., Chen C.Y. (2016). Intracerebroventricular urocortin 3 counteracts central acyl ghrelin-induced hyperphagic and gastroprokinetic effects via CRF receptor 2 in rats. Drug Des. Dev. Ther..

[28] Chen C.Y., Tsai C.Y., Lee W.J., Liaw W.J., Chiang C.H., Ho S.T., Lee S.D. (2012). Intracerebroventricular *O*-n-octanoylated ghrelin and its splice variant-induced feeding is blocked by insulin, independent of obestatin or CRF receptor, in satiated rats. Nutrition.

[29] Ting C.H., Chen Y.C., Liaw W.J., Lin H.C., Chen C.Y. (2016). Peripheral injection of pancreatic polypeptide enhances colonic transit without eliciting anxiety or altering colonic secretion in rats. Neuropeptides.

[30] Huang H.H., Chen L.Y., Doong M.L., Chang S.C., Chen C.Y. (2017). α-melanocyte stimulating hormone modulates the central acyl ghrelin-induced stimulation of feeding, gastrointestinal motility, and colonic secretion. Drug Des. Dev. Ther..

[31] Chen C.Y., Doong M.L., Li C.P., Liaw W.J., Lee H.F., Chang F.Y., Lin H.C., Lee S.D. (2010). A novel simultaneous measurement method to assess the influence of intracerebroventricular obestatin on colonic motility and secretion in conscious rats. Peptides.

[32] Chen C.Y., Chien E.J., Chang F.Y., Lu C.L., Luo J.C., Lee S.D. (2008). Impacts of peripheral obestatin on colonic motility and secretion in conscious fed rats. Peptides.

[33] Lawrence C.B., Snape A.C., Baudoin F.M., Luckman S.M. (2002). Acute central ghrelin and GH secretagogues induce feeding and activate brain appetite centers. Endocrinology.

[34] Nakazato M., Murakami N., Date Y., Kojima M., Matsuo H., Kangawa K., Matsukura S. (2001). A role for ghrelin in the central regulation of feeding. Nature.

[35] Wren A.M., Seal L.J., Cohen M.A., Brynes A.E., Frost G.S., Murphy K.G., Dhillo W.S., Ghatei M.A., Bloom S.R. (2001). Ghrelin enhances appetite and increases food intake in humans. J. Clin. Endocrinol. Metab..

[36] Druce M.R., Wren A.M., Park A.J., Milton J.E., Patterson M., Frost G., Ghatei M.A., Small C., Bloom S.R. (2005). Ghrelin increases food intake in obese as well as lean subjects. Int. J. Obes..

[37] Kojima M., Hosoda H., Date Y., Nakazato M., Matsuo H., Kangawa K. (1999). Ghrelin is a growth-hormone-releasing acylated peptide from stomach. Nature.

[38] Hosoda H., Kojima M., Matsuo H., Kangawa K. (2000). Ghrelin and des-acyl ghrelin: Two major forms of rat ghrelin peptide in gastrointestinal tissue. Biochem. Biophys. Res. Commun..

[39] Sato T., Nakamura Y., Shiimura Y., Ohgusu H., Kangawa K., Kojima M. (2012). Structure, regulation and function of ghrelin. J. Biochem..

[40] Yang J., Brown M.S., Liang G., Grishin N.V., Goldstein J.L. (2008). Identification of the acyltransferase that octanoylates ghrelin, an appetite-stimulating peptide hormone. Cell.

[41] Cornejo M.P., Castrogiovanni D., Schiöth H.B., Reynaldo M., Marie J., Fehrentz J.A., Perello M. (2019). Growth hormone secretagogue receptor signalling affects high-fat intake independently of plasma levels of ghrelin and LEAP2, in a 4-day binge eating model. J. Neuroendocrinol..

[42] Barrile F., M’Kadmi C., De Francesco P.N., Cabral A., García Romero G., Mustafá E.R., Cantel S., Damian M., Mary S., Denoyelle S. (2019). Development of a novel fluorescent ligand of growth hormone secretagogue receptor based on the N-Terminal Leap2 region. Mol. Cell Endocrinol..

[43] Shankar K., Metzger N.P., Singh O., Mani B.K., Osborne-Lawrence S., Varshney S., Gupta D., Ogden S.B., Takemi S., Richard C.P. (2021). LEAP2 deletion in mice enhances ghrelin’s actions as an orexigen and growth hormone secretagogue. Mol. Metab..

[44] Tezenas du Montcel C., Duriez P., Cao J., Lebrun N., Ramoz N., Viltart O., Gorwood P., Tolle V. (2023). The role of dysregulated ghrelin/LEAP-2 balance in anorexia nervosa. iScience.

[45] Mani B.K., Puzziferri N., He Z., Rodriguez J.A., Osborne-Lawrence S., Metzger N.P., Chhina N., Gaylinn B., Thorner M.O., Thomas E.L. (2019). LEAP2 changes with body mass and food intake in humans and mice. J. Clin. Investig..

[46] Sundaram S., Yan L. (2016). Time-restricted feeding reduces adiposity in mice fed a high-fat diet. Nutr. Res..

[47] Sorrell J., Yates E., Rivir M., Woods S.C., Hogenesch J.B., Perez-Tilve D. (2020). The central melanocortin system mediates the benefits of time-restricted feeding on energy balance. Physiol. Behav..

[48] Akamizu T., Takaya K., Irako T., Hosoda H., Teramukai S., Matsuyama A., Tada H., Miura K., Shimizu A., Fukushima M. (2004). Pharmacokinetics, safety, and endocrine and appetite effects of ghrelin administration in young healthy subjects. Eur. J. Endocrinol..

[49] Hagemann C.A., Jensen M.S., Holm S., Gasbjerg L.S., Byberg S., Skov-Jeppesen K., Hartmann B., Holst J.J., Dela F., Vilsbøll T. (2022). LEAP2 reduces postprandial glucose excursions and ad libitum food intake in healthy men. Cell Rep. Med..

[50] Hagemann C.A., Zhang C., Hansen H.H., Jorsal T., Rigbolt K.T.G., Madsen M.R., Bergmann N.C., Heimbürger S.M.N., Falkenhahn M., Theis S. (2021). Identification and Metabolic Profiling of a Novel Human Gut-derived LEAP2 Fragment. J. Clin. Endocrinol. Metab..

[51] Sutton E.F., Beyl R., Early K.S., Cefalu W.T., Ravussin E., Peterson C.M. (2018). Early Time-Restricted Feeding Improves Insulin Sensitivity, Blood Pressure, and Oxidative Stress Even without Weight Loss in Men with Prediabetes. Cell Metab..

[52] Ezpeleta M., Gabel K., Cienfuegos S., Kalam F., Lin S., Pavlou V., Song Z., Haus J.M., Koppe S., Alexandria S.J. (2023). Effect of alternate day fasting combined with aerobic exercise on non-alcoholic fatty liver disease: A randomized controlled trial. Cell Metab..

[53] Wilkinson M.J., Manoogian E.N.C., Zadourian A., Lo H., Fakhouri S., Shoghi A., Wang X., Fleischer J.G., Navlakha S., Panda S. (2020). Ten-Hour Time-Restricted Eating Reduces Weight, Blood Pressure, and Atherogenic Lipids in Patients with Metabolic Syndrome. Cell Metab..

[54] Rynders C.A., Thomas E.A., Zaman A., Pan Z., Catenacci V.A., Melanson E.L. (2019). Effectiveness of Intermittent Fasting and Time-Restricted Feeding Compared to Continuous Energy Restriction for Weight Loss. Nutrients.

