# Supplementary material for: Impacts of Central Administration of the Novel Peptide, LEAP-2, in Different Food Intake Models in Conscious Rats
Source: Nutrients. 2024 Jun 19;16(12):1946. doi: 10.3390/nu16121946 (PMC11206331; doi:10.3390/nu16121946)
Supplement: Supplementary file 1 [file nutrients-16-01946-s001.zip › nutrients-3021855-supplementary.pdf]

nutrients

MDPI

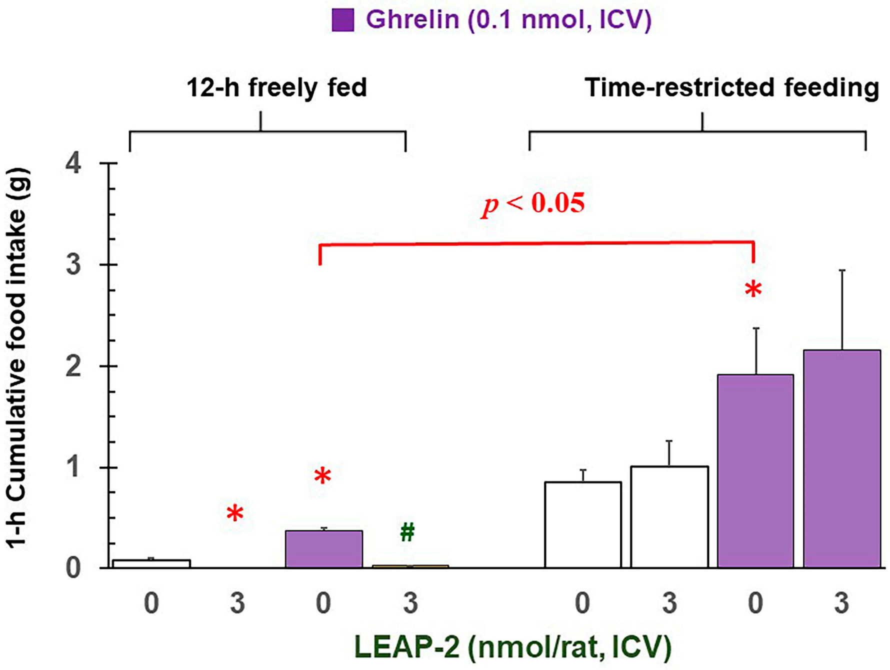

Figure S1. Comparison of 1 h cumulative food intake under 12 h freely fed and time restricted feeding states. The rats were administered with vehicle, *O*-*n*-octanoylated ghrelin (0.1 nmol/rat, ICV), LEAP-2 (3 nmol/rat, ICV), and *O*-*n*-octanoylated ghrelin (0.1 nmol/rat, ICV) + pre-treatment with LEAP-2 (3 nmol/rat, ICV). The sample size for each experimental group under each feeding state was 12 rats. LEAP-2: liver-expressed antimicrobial peptide-2; ICV: intracerebroventricular injection. *$p < 0.05$ vs. vehicle + vehicle, #$p < 0.05$ vs. vehicle + ghrelin.

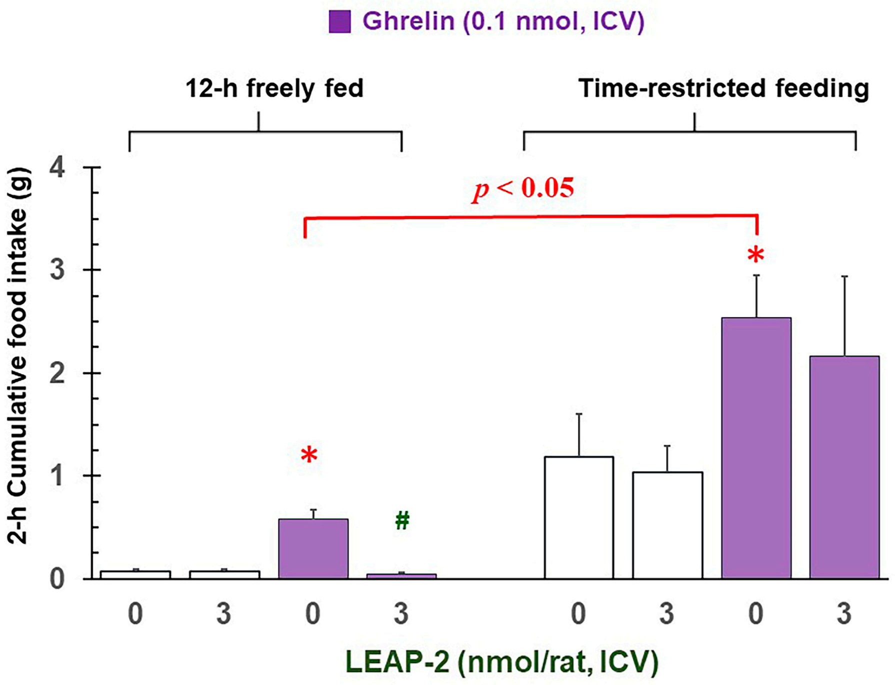

Figure S2. Comparison of 2 h cumulative food intake under 12 h freely fed and time restricted feeding states. The rats were administered with vehicle, *O*-*n*-octanoylated ghrelin (0.1 nmol/rat, ICV), LEAP-2 (3 nmol/rat, ICV), and *O*-*n*-octanoylated ghrelin (0.1 nmol/rat, ICV) + pre-treatment with LEAP-2 (3 nmol/rat, ICV). The sample size for each experimental group under each feeding state was 12 rats. LEAP-2: liver-expressed antimicrobial peptide-2; ICV: intracerebroventricular injection. *$p < 0.05$ vs. vehicle + vehicle, #$p < 0.05$ vs. vehicle + ghrelin.

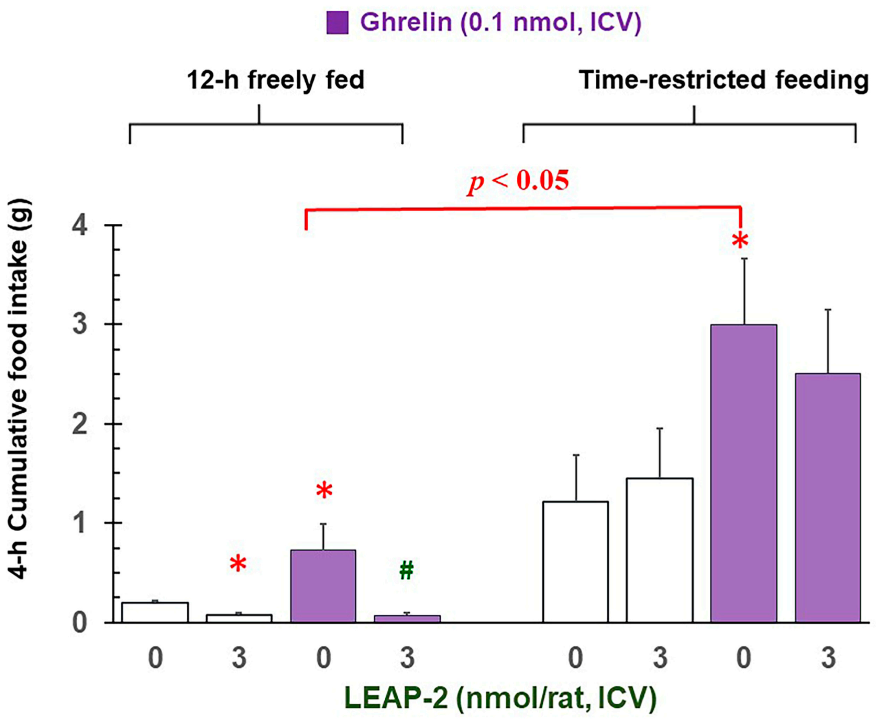

Figure S3. Comparison of 4 h cumulative food intake under 12 h freely fed and time restricted feeding states. The rats were administered with vehicle, *O-n*-octanoylated ghrelin (0.1 nmol/rat, ICV), LEAP-2 (3 nmol/rat, ICV), and *O-n*-octanoylated ghrelin (0.1 nmol/rat, ICV) + pre-treatment with LEAP-2 (3 nmol/rat, ICV). The sample size for each experimental group under each feeding state was 12 rats. LEAP-2: liver-expressed antimicrobial peptide-2; ICV: intracerebroventricular injection. *$p < 0.05$ vs. vehicle + vehicle, #$p < 0.05$ vs. vehicle + ghrelin.
